# Adenomatoid tumour of the liver

**DOI:** 10.1136/jcp.2007.054684

**Published:** 2008-05-27

**Authors:** S Nagata, S Aishima, K Fukuzawa, H Takagi, H Yonemasu, Y Iwashita, T Kinoshita, K Wakasugi

**Affiliations:** 1 Department of Surgery, Nakabaru Hospital, Fukuoka, Japan; 2 Department of Anatomic Pathology, Hamanomachi Hospital, Fukuoka, Japan; 3 Department of Surgery, Oita Red Cross Hospital, Oita, Japan; 4 Department of Roentgenology, Oita Red Cross Hospital, Oita, Japan; 5 Department of Anatomic Pathology, Oita Red Cross Hospital, Oita, Japan

## Abstract

An unusual primary adenomatoid tumour arising in the normal liver is described. Hepatectomy was performed, and the patient is alive and free of disease 1 year postsurgery. Grossly, the tumour showed a haemorrhagic cut surface with numerous microcystic structures. Histological examination revealed cystic or angiomatoid spaces of various sizes lined by cuboidal, low-columnar, or flattened epithelioid cells with vacuolated cytoplasm and round to oval nuclei. The epithelioid cells were entirely supported by proliferated capillaries and arteries together with collagenous stroma. Immunohistochemical studies showed that the epithelioid cells were strongly positive for a broad spectrum of cytokeratins (AE1/AE3, CAM5.2, epithelial membrane antigen and cytokeratin 7) and mesothelial markers (calretinin, Wilms’ tumour 1 and D2-40). These cells were negative for Hep par-1, carcinoembryonic antigen, neural cell adhesion molecule, CD34, CD31 and HMB45. Atypically, abundant capillaries were observed; however, the cystic proliferation of epithelioid cells with vacuoles and immunohistochemical profile of the epithelioid element were consistent with hepatic adenomatoid tumour.

**Take-home messages**

- This is believed to be the first case report describing a solitary hepatic adenomatoid tumour.
- The hepatic adenomatoid tumour showed prominent cystic changes with an abundant vascular component, and immunohistochemical studies showed that it reacted with a broad spectrum of cytokeratins and mesothelial markers.
- When a hypervascular hepatic tumour is detected, adenomatoid tumour needs to be considered in the differential diagnosis.

Adenomatoid tumours are benign neoplasms of mesothelial origin and are most commonly encountered in the testis, epididymis, fallopian tube, uterus and ovary.^1^^–^3 Sporadic cases have been reported in the adrenal glands,^4^ pleura^5^ and pancreas.^6^ Recently, multiple adenomatoid tumours involving the liver and peritoneum have been reported;^7^ however, there have been no reports of a solitary hepatic adenomatoid tumour.

We report the case of hypervascular hepatic adenomatoid tumour incidentally discovered and diagnosed based on the findings of light microscopy and immunohistochemistry.

## CASE PRESENTATION

### Clinical findings

A 39-year-old man without any remarkable previous medical history was introduced to our unit from another hospital for the treatment of a 2.0 cm hypervascular hepatic tumour in segment V; the tumour had been recently discovered by abdominal ultrasonography (US) performed during a general check-up. Physical examination was normal, and his carcinoembryonic antigen (CEA), carbohydrate antigen 19-9, α-fetoprotein, and protein induced by vitamin K absence-II levels were within the normal range. The patient was negative for both the hepatitis B virus surface antigen and the hepatitis C virus antibody.

**Figure 1:**
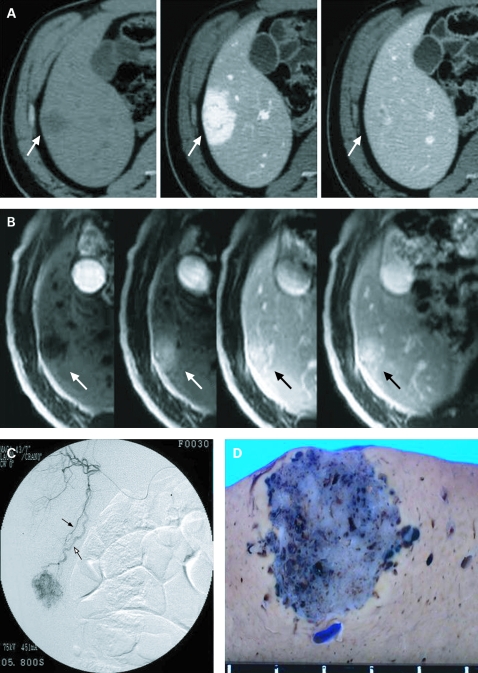
(A) CT revealed that the tumour was hypointense pre-enhancement and highly enhanced in the arterial phase with early washout (arrows). (B) Enhanced MRI after gadolinium injection. The tumour was hyperintense on T1-weighted images in the arterial phase but demonstrated prolonged enhancement in the portal venous and delayed phases (arrows). (C) Angiography showed a definite tumour with an arterioportal shunt (the artery (A5, filled arrow) and the portal vein (P5, open arrow)). (D) Grossly, the 1.5 cm soft mass in the subcapsular region showed a haemorrhagic cut surface with numerous microcystic spongiotic structures but was non-encapsulated.

Abdominal US demonstrated a hyperechogenic round tumour in hepatic segment V. Abdominal CT demonstrated that the tumour was hypointense in pre-enhancement and highly enhanced in the arterial phase with early washout (fig 1A). In MRI, the tumour was hypointense on T1-weighted images and highly hyperintense on T2-weighted images. After gadolinium injection, the tumour was hyperintense on T1-weighted images in the arterial phase but demonstrated prolonged enhancement in the portal venous and delayed phases, probably suggesting the presence of fibrous tissue (fig 1B). Angiography demonstrated the tumour as a hypervascular mass fed by A5 from an arterioportal shunt (fig 1C). Hepatic segmentectomy was performed because of the possibility of malignancy and because the patient consented to surgery and not liver biopsy or observation. The patient is alive and free of disease 1 year postsurgery.

### Pathological findings

Grossly, the 1.5 cm soft mass in the subcapsular region showed a haemorrhagic cut surface with numerous microcystic structures and was non-encapsulated (fig 1D).

**Figure 2:**
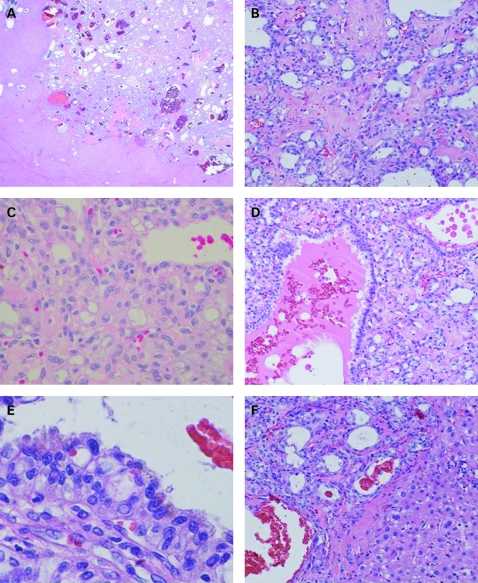
Histological findings by H&E staining. (A) The well-circumscribed nodular tumour comprising cystic spaces. (B) Cystic spaces of various sizes supported by collagenous stroma. (C) The cystic or angiomatoid spaces are lined with cuboidal, low-columnar or flattened epithelioid cells, and contain abundant red blood cells and colloid-like materials (D). (E) The epithelioid cells show small- to large-sized vacuoles or eosinophilic cytoplasm with round to oval nuclei. (F) The border of the tumour is surrounded by fine collagenous bands, and neither epithelioid cells nor stromal cells infiltrated the liver parenchyma.

Histologically, the well-circumscribed nodular tumour comprised cystic spaces of various sizes with collagenous stroma (fig 2A, B). The cystic or angiomatoid spaces were lined with cuboidal, low-columnar or flattened epithelioid cells, and contained abundant red blood cells and colloid-like materials (fig 2C, D). The epithelioid cells showed small- to large-sized vacuoles or eosinophilic cytoplasm with round-to-oval nuclei (fig 2E). The tumour border was surrounded by fine collagenous bands without invasion into the liver parenchyma (fig 2F). The presence of mitotic figures, definite cellular atypia or an invasive growth pattern was not recognised.

**Figure 3:**
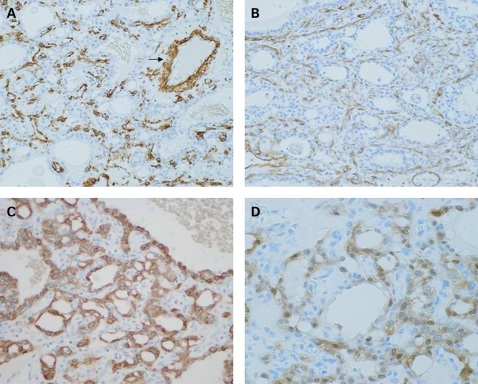
Histological findings by immunohistochemical staining. (A) Non-epithelioid cells are diffusely positive for α-smooth muscle actin and show the arterial wall (arrow). (B) CD31-positive capillaries surround the epithelioid cells. The epithelioid cells are diffusely positive for AE1/AE3 (C), and calretinin (D).

Immunohistochemically, the non-epithelioid cells were diffusely positive for α-smooth muscle actin (Sigma-Aldrich, St Louis, Missouri, USA; fig 3A), CD31 (Dako, Glostrup, Denmark; fig 3B) and CD34 (Novocastra, Newcastle, UK). These features clearly indicated that the cystic structure of the epithelioid cells was entirely supported by myofibroblastic cells, capillaries and arteries. The epithelioid cells were diffusely positive for pancytokeratin, AE1/AE3 (Labvision, Fremont, California, USA; fig 3C), epithelial membrane antigen (EMA; Dako), CAM5.2 (Becton Dickinson, San Jose, California, USA), cytokeratin (CK) 7 (Dako) and CK19 (Dako), as well as for the mesothelial markers calretinin (Zymed, San Francisco, California, USA; fig 3D), Wilms’ tumour 1 (WT49; Novocastra) and D2-40 (Nichirei Bioscience, Tokyo, Japan). The epithelioid cells were negative for Hep par-1 (Dako), carcinoembryonic antigen (Zymed), neural cell adhesion molecule (NCAM; Novocastra Laboratories), CD31, CD34 and HMB45 (Enzo, Farmingdale, New York, USA).

## DISCUSSION

We describe an unusual case of a hepatic adenomatoid tumour that was incidentally detected by radiography. Thorough clinical and radiological examinations did not reveal any evidence of another tumour. During preoperative diagnosis of the hypervascular hepatic mass, we suspected haemangioma or haemangioma-like tumour, epithelioid haemangioendothelioma and haemangiopericytoma, both of which occasionally demonstrate malignant characteristics. Hypervascular hepatic tumours such as hepatocellular carcinoma, haemangioma, focal nodular hyperplasia, hepatic adenoma and hepatic angiomyolipoma were also included in the differential diagnosis.^8^^–^10 However, the possibility of hepatocellular carcinoma was undeniable, and surgery was performed.

Grossly, the tumour resembled a haemangioma with regard to blood-related features and cystic, spongiotic appearance. However, the microscopic features of the tumour in our case were not consistent with those of any of the above-mentioned tumours. Neither hepatocellular proliferation nor simple endothelial proliferation was observed. This tumour was a soft, well-circumscribed, but non-capsulated mass with a prominently haemorrhagic and multicystic surface. Histologically, the tumour comprised cuboidal or flattened epithelioid cells arranged in a cystic or angiomatoid structure, supported by prominent capillaries and arteries. These epithelioid cells, which did not look like bile duct, were positive for a broad spectrum of cytokeratins such as AE1/AE3, CAM5.2, EMA, CK7 and CK19. Additional staining of our specimen for the mesothelial markers calretinin and D2-40 suggested the mesothelial origin of the epithelioid cells of the tumour. Isotalo *et al.* reported that five adenomatoid tumours of the adrenal gland were strongly positive for CK7.^4^

Several common histological patterns have been observed in adenomatoid tumours, including adenomatoid, cystic, angiomatoid, tubular, plexiform and canalicular patterns. A combination of tubular and angiomatoid patterns is more commonly observed. Prominent cystic changes were observed in our case. The cystic type of adenomatoid tumour has been reported to arise in the uterus,^1^ ovary^3^ and pancreas.^6^ In previous reports, adrenal^4^ and pleural^5^ lesions have shown an infiltrative growth pattern, but the pancreatic adenomatoid tumour was surrounded by a distinct fibrous rim, similar to our tumour that presented with a thin fibrous rim along the border. Therefore, the morphological characteristics of cystic appearance and circumscribed border observed in our case are rare features. The presence of cytoplasmic vacuoles and red blood cells indicated hepatic epithelioid haemangioendothelioma;^8^ however, the epithelioid cells were negative for endothelial markers and no invasive growth pattern was detected. Hepatic adenomatoid tumour has been recently reported as multiple tumours of the liver and peritoneum;^7^ however, a solitary hepatic adenomatoid tumour has not been reported. Although the origin of the tumour in our case remains uncertain, based on its location in the subcapsular region of the liver, we believe that it arose from the mesothelial cells of the liver surface.

An abundant vascular component has rarely been observed in typical adenomatoid tumours, but a vascularised component is present in some ovarian adenomatoid tumours.^3^ In the present case, the mechanisms of vascular proliferation and accumulation of red blood cells in the cystic spaces were unknown, but the many vascular spaces indicated the predominantly vascularised character and raised the possibility of an other neoplasm or tumour-like conditions.

Immunohistochemically, the epithelioid cells reacted with a broad spectrum of cytokeratins and calretinin. Based on the morphological features and immunohistochemical profile, we believe that the characteristics of the hypervascular liver tumour are consistent with those of an adenomatoid tumour. Therefore, when a small-sized hypervascular tumour located in the subcapsular region is detected, the diagnosis of adenomatoid tumour should be considered.
